# Peripheral blood metabolic profiles of chronic rhinosinusitis and their mediating role between obesity and disease

**DOI:** 10.1186/s12944-025-02672-w

**Published:** 2025-07-28

**Authors:** Zengxiao Zhang, Shunke Li, Shizhe Zhou, Lin Wang, Xudong Yan, Longgang Yu, Yan Jiang

**Affiliations:** 1 Department of Otorhinolaryngology Head and Neck Surgery, the Affiliated Hospital of Qingdao University, No. 59, Haier Road, Laoshan District, Qingdao, 266003 China; 2 Department of Endocrinology and Metabolism, Beijing Tsinghua Changgung Hospital, Tsinghua University, Beijing, China

**Keywords:** Body mass index, Eosinophilia, Inflammation, Metabolism, Rhinosinusitis

## Abstract

**Background:**

Chronic rhinosinusitis (CRS) is increasingly linked to systemic inflammation, however, research on peripheral blood metabolic patterns in CRS patients remains limited. This study aimed to investigate peripheral blood metabolic profiles in eosinophilic CRS and non-eosinophilic CRS, while exploring the mediating role of metabolites in the relationship between body mass index and CRS.

**Methods:**

Clinical data were collected from 1,151 CRS patients and 814 healthy controls, classifying patients into eosinophilic CRS and non-eosinophilic CRS groups based on tissue eosinophil counts. Peripheral blood metabolic profiles were compared across different CRS endotypes and between CRS patients and healthy controls. Causal mediation analysis assessed the mediating effects of metabolites on the relationship between body mass index and CRS.

**Results:**

CRS patients exhibited distinct metabolic profiles, with dysregulated lipid metabolism characterized by increased triglycerides, free fatty acids, and lipoprotein(a), but patients with eosinophilic CRS had higher triglycerides, while non-eosinophilic CRS had higher free fatty acids. Cystatin-C effectively differentiated CRS endotypes (area under the curve = 0.735). Elevated body mass index was a risk factor for both eosinophilic CRS and non-eosinophilic CRS patients, with peripheral free fatty acids and Cystatin-C mediating this effect.

**Conclusions:**

This study reveals distinct metabolic profiles in patients with CRS, supporting its link to systemic inflammation. Promoting healthy dietary habits and weight control is therefore a cornerstone of sustainable, preventive care, offering a practical strategy to improve long-term patient well-being, particularly in refractory cases.

**Supplementary Information:**

The online version contains supplementary material available at 10.1186/s12944-025-02672-w.

## Introduction

Chronic rhinosinusitis (CRS) is defined by sustained inflammation within the sinonasal mucosa. As a complex and heterogeneous condition, it imposes significant social and economic burdens [1, 2], with a self-reported prevalence ranging from 10 to 28% [3, 4]. Current studies have progressively acknowledged the link between CRS and systemic inflammation, and preliminary investigations have suggested a relationship between metabolic syndrome and CRS [5, 6]. Nevertheless, research investigating the variations in peripheral blood metabolic profiles between patients with CRS and healthy individuals remains markedly limited.

CRS can be categorized into different endotypes according to tissue eosinophil counts: eosinophilic CRS (eCRS) and non-eosinophilic CRS (non-eCRS). The two endotypes exhibit unique patterns of inflammation as well as clinical characteristics, drawing considerable interest from rhinologists and immunologists [7–9]. Exploring the differences in peripheral blood metabolic profiles between these endotypes is crucial for understanding their systemic metabolic patterns and identifying potential blood markers for predicting CRS endotypes.

Previous studies have documented the association between obesity and CRS [10–12], and in a prior Mendelian randomization (MR) study [13], a unidirectional causal effect of body mass index (BMI) on CRS was identified. However, the mechanisms underlying this relationship remain unclear; furthermore, limitations in the available data of genome-wide association studies prevented the differentiation between CRS endotypes in that study. Therefore, this study was designed to assess differences in BMI among the CRS endotypes and healthy populations, and investigating whether blood metabolites mediate the effect of BMI on these endotypes.

The study aimed to compare peripheral blood metabolic profiles between patients with eCRS, non-eCRS, and healthy controls, revealing the systemic metabolic patterns of each endotype. Additionally, as a key novel aspect of this study, the investigation explored the predictive value of blood metabolic indices for CRS endotypes and evaluated the mediating effects of peripheral blood metabolites in the relationship between BMI and CRS endotypes using causal mediation analysis, further elucidating the intrinsic mechanisms linking BMI and CRS.

## Methods

### Study population

This study was granted by the institutional Ethics Review Board (ID: QYFY WZLL 28781). As this study is a retrospective analysis utilizing anonymized clinical data from medical records, informed consent was not required.

Patients with CRS were included from the hospital’s pathology database between January 2013 and June 2023 based on eligibility criteria.

The inclusion criteria were as follows: (1) Adult patients with CRS confirmed histopathologically and in accordance with the European Position Paper on Rhinosinusitis and Nasal Polyps (EPOS) 2020 [3]; (2) those who underwent peripheral blood metabolic profiles within 1 week prior to nasal endoscopic surgery; (3) those who had complete demographic and clinical information and peripheral blood metabolic indices.

The exclusion criteria were as follows: (1) Patients who indicated co-morbidity with other sinonasal diseases such as fungal rhinosinusitis, cystic fibrosis, ciliary dyskinesia, and sinonasal neoplasms; (2) co-morbid immunodeficiency; (3) pregnant; (4) co-morbid diseases affecting blood metabolic profiles (e.g., cardiovascular, cerebrovascular, chronic liver/kidney diseases, diabetes, malignancies); (5) recent use of medications affecting blood metabolic profiles (within 1 month), such as systemic corticosteroids.

Healthy controls were obtained from adults attending the Health Management Center of our hospital for physical examination during the same period and underwent peripheral blood metabolic profiles. Participants were excluded if any of the following conditions were present: (1) Individuals who indicated co-morbidity with sinonasal diseases such as CRS, fungal rhinosinusitis, allergic rhinitis, and sinonasal neoplasms, etc.; exclusion criteria (2)-(6) for healthy controls were the same as exclusion criterion (2)-(6) for patients with CRS.

### Data collection

Demographic and clinical information was extracted separately for both patients with CRS and healthy controls, including gender, age, BMI, smoking and alcohol dependence, and clinical diagnosis, etc. Smoking dependence was defined as individuals who had smoked more than 100 cigarettes in their lifetime and who still reported smoking every day or on some days [14, 15]. Alcohol dependence was defined as meeting at least the National Health Interview Survey (NHIS) criteria for a moderate drinker (> 3 drinks/week) [16]. One alcoholic drink-equivalent was defined as a drink containing 14 g of pure alcohol (approximately 0.6 fluid ounces or 1.2 tablespoons). This was equivalent to a 12-ounce serving of beer (5% alcohol), a 5-ounce glass of wine (12% alcohol), or a 1.5-ounce shot of distilled spirits (40% alcohol).

Peripheral blood metabolic profiles levels were collected, including triglyceride (TG), free fatty acid (FFA), total cholesterol (TC), low-density lipoprotein (LDL), high-density lipoprotein (HDL), lipoprotein(a) (LP(a)), albumin (ALB), globulin (GLB), prealbumin (PAB), direct bilirubin (DBIL), indirect bilirubin (IBIL), alanine aminotransferase (ALT), aspartate aminotransferase (AST), gamma-glutamyl transferase (GGT), lactate dehydrogenase (LDH), leucine aminopeptidase (LAP), adenosine deaminase (ADA), sialic acid (SA), creatinine (Crea), cystatin-C (Cys-C), C1q, glucose, and uric acid (UA).

### Histologic typing

Surgically collected tissues of nasal polyps/ethmoidal sinus mucosa were evaluated by a senior pathologist under high-powered microscope fields (HPFs, 400×) after H&E staining. Five HPFs were randomly selected for eosinophil counting and averaged. Tissue eosinophil counts of at least 55/HPF were defined as eCRS and < 55/HPF were defined as non-eCRS [17].

### Study design

An initial comparison of demographic data and peripheral blood metabolic profiles was performed between the CRS group and healthy controls. Subsequently, the CRS group was stratified into the eCRS and non-eCRS subgroups for comparison. Independent predictors for CRS endotypes were identified using univariate and multivariate logistic regression, with receiver operating characteristic (ROC) curves evaluating their predictive value. Furthermore, subgroup analyses between eCRS/non-eCRS and healthy controls were conducted; blood metabolites that were significant in univariate logistic regression indicated that they were associated with eCRS/non-eCRS, and these metabolites were considered potential mediating variables; causal mediation analysis was applied to elucidate the mediating effects of these metabolites between BMI and eCRS/non-CRS.

### Statistical analysis

Statistical Package for the Social Sciences version 27.0 (IBM, Corp, Armonk, NY, USA) was applied for statistical analysis. Normality tests were performed for quantitative data. Quantitative data of normal distribution were demonstrated using mean ± standard deviation ($\:\stackrel{-}{x}$±*s*), and Student’s t-tests were used for comparisons between groups. Median ± interquartile range (*M* ± *Q*_*R*_) was used to determine quantitative data of non-normal distribution, and Mann–Whitney U tests were used for comparisons between groups. Frequencies and proportion were used to determine qualitative data; the χ^2^ test was used for comparisons between groups. Variables associated with CRS were evaluated using logistic regression, and odds ratios (ORs) and 95% confidence intervals (CIs) were calculated.

The “pROC” package of R software version 4.3.3 (R Foundation, Vienna, Austria) was performed for generating ROC curves to evaluate the ability of blood metabolism indices to differentiate eCRS and non-eCRS; the “tidyverse”, “broom”, and “ggdist” packages were performed for generating fitted curves to evaluate the association between BMI and eCRS/non-eCRS.

The “mediation” package of R software version 4.3.3 (R Foundation, Vienna, Austria) was performed for causal mediation analysis; the simulations were repeated 5000 times using the bootstrap method to estimate the confidence intervals for the mediating effects. In causal mediation analysis, the natural indirect effect, which is the effect of the mediator on the outcome when exposure is being controlled, is defined as the mediating effect; this method is based on the counterfactual framework, which is capable of overcoming the effects of interactions between variables well and obtaining reliable inferences about causal mediation [18, 19].

All statistical analyses were conducted using two-tailed tests, with the threshold for significance established at *P* < 0.05.

## Results

### Demographics and peripheral blood metabolic profiles of the study population

This study enrolled 1965 subjects, comprising 1151 patients diagnosed with CRS and 814 healthy controls.

**Fig. 1:**
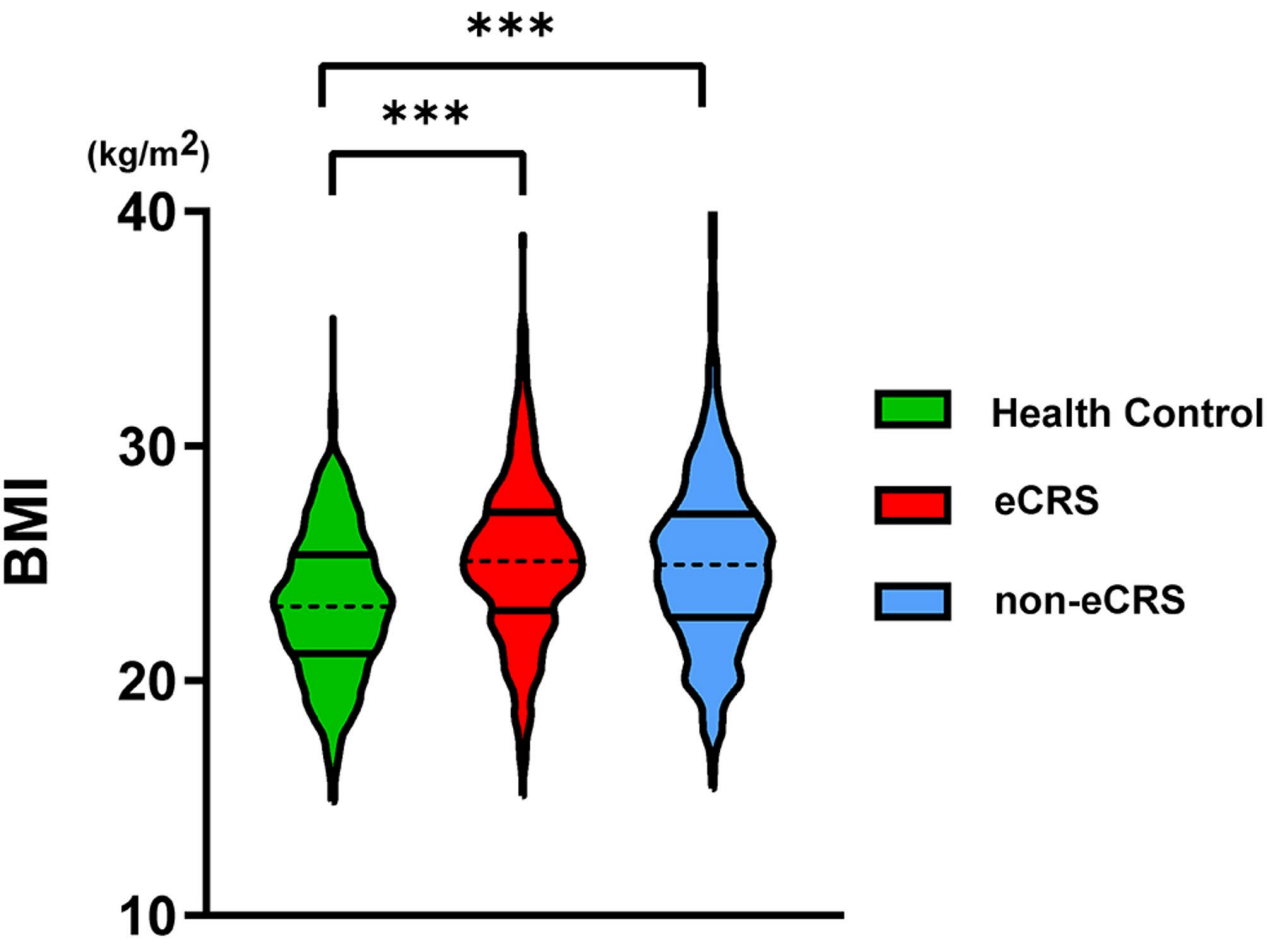
Comparison of BMI among healthy controls, eCRS patients, and non-eCRS patients. BMI, body mass index; eCRS, eosinophilic chronic rhinosinusitis; non-eCRS, non-eosinophilic chronic rhinosinusitis

As shown in Table 1, patients with CRS and healthy controls did not differ significantly with respect to age, smoking dependence, or alcohol dependence (all *P* > 0.05). However, the CRS group was composed of a significantly larger proportion of males (*P* < 0.001) and also exhibited a higher BMI (*P* < 0.001) relative to the healthy controls. The difference and distribution of BMI among healthy controls, and patients with different CRS endotypes is provided in Fig. 1.

**Table 1:** Baseline characteristics of the CRS patients and healthy controls

	CRS patients (*n* = 1151)	Health controls (*n* = 814)	*P*
Gender, male	794 (68.98%)	430 (52.83%)	< 0.001
Age, years^*^	52.00 ± 20.00	51.00 ± 14.00	0.306
BMI, kg/m^2*^	25.00 ± 4.50	23.16 ± 4.22	< 0.001
Smoking dependence	431 (37.45%)	304 (37.35%)	0.964
Alcohol dependence	457 (39.70%)	311 (38.21%)	0.503

^*^ *M* ± *Q*_*R*_; BMI, body mass index; CRS, chronic rhinosinusitis

Table 2 presents the peripheral blood metabolic profiles of the two groups. Compared with the healthy controls, patients in the CRS group exhibited significantly higher levels of TG, FFA, LP(a), Crea, Cys-C, and UA, in contrast to significantly lower levels of LDH (all *P* < 0.05). Conversely, no significant inter-group distinctions were observed for TC, LDL, HDL, ALB, GLB, PAB, DBIL, IBIL, ALT, AST, GGT, LAP, ADA, SA, C1q, or glucose (all *P* > 0.05).

**Table 2:** Metabolic characteristics of peripheral blood between CRS patients and healthy controls

	CRS patients (*n* = 1151)	Health controls (*n* = 814)	*P*
TG, mmol/L^*^	1.15 ± 0.92	1.04 ± 0.94	< 0.001
FFA, mmol/L^*^	0.44 ± 0.29	0.31 ± 0.27	< 0.001
TC, mmol/L^*^	4.88 ± 1.43	4.89 ± 1.37	0.779
LDL, mmol/L^*^	2.84 ± 1.07	2.86 ± 1.01	0.190
HDL, mmol/L^*^	1.40 ± 0.44	1.41 ± 0.46	0.774
LP(a), mg/L^*^	126.70 ± 161.25	110.00 ± 129.25	< 0.001
ALB, g/L^*^	48.30 ± 12.80	48.56 ± 13.00	0.054
GLB, g/L^*^	30.80 ± 8.25	30.46 ± 8.92	0.098
PAB, mg/L^*^	290.60 ± 95.28	283.90 ± 85.40	0.081
DBIL, µmol/L^*^	3.60 ± 2.17	3.58 ± 2.09	0.413
IBIL, µmol/L^*^	9.90 ± 4.98	10.02 ± 4.67	0.832
ALT, U/L^*^	19.00 ± 13.10	19.70 ± 13.45	0.177
AST, U/L^*^	18.00 ± 7.00	18.50 ± 6.75	0.070
GGT, U/L^*^	19.00 ± 18.00	18.60 ± 6.00	0.469
LDH, U/L^*^	163.00 ± 37.00	170.00 ± 39.00	< 0.001
LAP, U/L^*^	42.00 ± 9.60	42.00 ± 11.30	0.163
ADA, U/L^*^	9.00 ± 3.00	9.00 ± 3.00	0.375
SA, mg/L^*^	64.00 ± 420.81	62.94 ± 436.50	0.219
Crea, µmol/L^*^	80.30 ± 25.00	78.00 ± 24.33	< 0.001
Cys-C, mg/mL^*^	840 ± 170	730 ± 180	< 0.001
C1q, mg/L^*^	177.00 ± 47.40	177.00 ± 47.90	0.928
Glucose, mmol/L^*^	4.98 ± 0.87	4.95 ± 0.83	0.474
UA, µmol/L^*^	338.00 ± 122.00	296.00 ± 125.00	< 0.001

^*^ *M* ± *Q*_*R*_; ADA, adenosine deaminase; ALB, albumin; ALT, alanine aminotransferase; AST, aspartate aminotransferase; Crea, creatinine; CRS, chronic rhinosinusitis; Cys-C, cystatin C; DBIL, direct bilirubin; FFA, free fatty acid; GGT, gamma-glutamyl transferase; GLB, globulin; HDL, high-density lipoprotein; IBIL, Indirect bilirubin; LAP, leucine aminopeptidase; LDH, lactate dehydrogenase; LDL, low-density lipoprotein; LP(a), lipoprotein(a); PAB, prealbumin; SA, sialic acid; TC, total cholesterol; TG, triglyceride; UA, uric acid

As shown in Table S1, univariate logistic regression identified significant associations between CRS and gender, BMI, TG, FFA, LP(a), LDH, Crea, Cys-C, and UA (all *P* < 0.05). In multivariate analysis, with adjustment for age, smoking dependence, and alcohol dependence, the results showed that gender (OR [95% CI]: 2.333 [1.678–3.245]; *P* < 0.001), BMI (1.156 [1.116–1.197]; *P* < 0.001), TG (0.843 [0.750–0.947]; *P* = 0.004), FFA (19.786 [11.557–33.874]; *P* < 0.001), LP(a) (1.001 [1.000–1.002]; *P* < 0.001), LDH (0.989 [0.985–0.992]; *P* < 0.001), and Cys-C (1.004 [1.003–1.005]; *P* < 0.001) were still independently associated with CRS.

The logistic regression curve indicated a positive dose-response relationship between BMI and the risk of CRS (Figure S1).

### Comparison of peripheral blood metabolic profiles between eCRS and non-eCRS groups

Patients with CRS were further divided into the eCRS group of 335 patients and the non-eCRS group of 816 patients according to endotypes. Table 3 shows that there was no significant difference in BMI and alcohol dependence between the two groups (all *P* > 0.05). However, eCRS patients were older (*P* = 0.012) and had a higher proportion of males (*P* < 0.001) and smokers (*P* < 0.001).

**Table 3:** Baseline characteristics of the eCRS patients and non-eCRS patients

	eCRS patients (*n* = 335)	non-eCRS patients (*n* = 816)	*P*
Gender, male	255 (76.12%)	539 (66.05%)	< 0.001
Age, years^*^	53.00 ± 18.00	52.00 ± 22.00	0.012
BMI, kg/m^2*^	25.20 ± 4.30	24.95 ± 4.48	0.274
Smoking dependence	150 (44.78%)	281 (34.44%)	< 0.001
Alcohol dependence	147 (43.88%)	310 (37.99%)	0.064

^*^ *M* ± *Q*_*R*_; BMI, body mass index; eCRS, eosinophilic chronic rhinosinusitis; non-eCRS, non-eosinophilic chronic rhinosinusitis

Table 4 details the comparison of metabolic profiles between the eCRS and non-eCRS patients. Patients in the eCRS group had high levels of TG (*P* = 0.005), IBIL (*P* = 0.042), SA (*P* = 0.043), Crea (*P* < 0.001), Cys-C (*P* < 0.001), and UA (*P* < 0.001), but lower FFA (*P* < 0.001) and glucose (*P* = 0.004) compared with the non-eCRS group. For the remaining metabolites, no significant distinctions were found between between the two groups (*P* > 0.05).

**Table 4:** Metabolic characteristics of peripheral blood between eCRS patients and non-eCRS patients

	eCRS patients (*n* = 335)	non-eCRS patients (*n* = 816)	*P*
TG, mmol/L^*^	1.29 ± 1.00	1.11 ± 0.86	0.005
FFA, mmol/L^*^	0.40 ± 0.23	0.48 ± 0.30	< 0.001
TC, mmol/L^*^	4.86 ± 1.41	4.88 ± 1.45	0.409
LDL, mmol/L^*^	2.86 ± 1.10	2.83 ± 1.04	0.302
HDL, mmol/L^*^	1.37 ± 0.41	1.40 ± 0.45	0.137
LP(a), mg/L^*^	128.00 ± 152.00	125.00 ± 174.40	0.972
ALB, g/L^*^	47.77 ± 13.60	48.52 ± 12.36	0.911
GLB, g/L^*^	30.50 ± 8.59	30.88 ± 8.24	0.153
PAB, mg/L^*^	287.80 ± 97.20	292.30 ± 93.22	0.657
DBIL, µmol/L^*^	3.70 ± 2.26	3.60 ± 2.09	0.097
IBIL, µmol/L^*^	10.00 ± 4.88	9.84 ± 4.98	0.042
ALT, U/L^*^	19.10 ± 14.00	19.00 ± 13.40	0.569
AST, U/L^*^	18.00 ± 6.80	18.00 ± 7.00	0.563
GGT, U/L^*^	19.00 ± 18.00	19.00 ± 18.50	0.891
LDH, U/L^*^	163.00 ± 36.00	163.00 ± 36.80	0.782
LAP, U/L^*^	41.90 ± 8.20	42.00 ± 10.68	0.679
ADA, U/L^*^	9.00 ± 4.00	9.00 ± 4.00	0.051
SA, mg/L^*^	66.09 ± 436.97	63.48 ± 403.09	0.043
Crea, µmol/L^*^	84.30 ± 22.00	79.00 ± 26.00	< 0.001
Cys-C, mg/mL^*^	910 ± 180	800 ± 180	< 0.001
C1q, mg/L^*^	173.00 ± 50.50	178.00 ± 45.45	0.104
Glucose, mmol/L^*^	4.90 ± 0.77	5.03 ± 0.89	0.004
UA, µmol/L^*^	355.20 ± 127.00	326.00 ± 119.52	< 0.001

^*^ *M* ± *Q*_*R*_; ADA, adenosine deaminase; ALB, albumin; ALT, alanine aminotransferase; AST, aspartate aminotransferase; Crea, creatinine; Cys-C, cystatin C; DBIL, direct bilirubin; eCRS, eosinophilic chronic rhinosinusitis; FFA, free fatty acid; GGT, gamma-glutamyl transferase; GLB, globulin; HDL, high-density lipoprotein; IBIL, Indirect bilirubin; LAP, leucine aminopeptidase; LDH, lactate dehydrogenase; LDL, low-density lipoprotein; LP(a), lipoprotein(a); non-eCRS, non-eosinophilic chronic rhinosinusitis; PAB, prealbumin; SA, sialic acid; TC, total cholesterol; TG, triglyceride; UA, uric acid

In the univariate logistic regression (Table 5), a significant association with eCRS was found for gender (OR [95% CI]: 1.638 [1.226–2.189], *P* < 0.001), age (1.003 [1.003–1.022], *P* = 0.009), smoking dependence (1.544 [1.191–2.001], *P* = 0.001), TG (1.220 [1.078–1.380], *P* < 0.001), FFA (0.315 [0.170–0.584], *P* < 0.001), SA (1.001 [1.000–1.002], *P* < 0.001), Crea (1.016 [1.008–1.024], *P* < 0.001), Cys-C (1.008 [1.006–1.009], *P* < 0.001), glucose (0.819 [0.704–0.952], *P* = 0.009), UA (1.002 [1.001–1.004], *P* = 0.009) were associated with eCRS. The above associated variables were entered into a multivariate logistic regression model, with adjustment for BMI and alcohol dependence as covariates, the results showed that FFA (OR [95% CI]: 0.306 [0.142–0.658], *P* = 0.002), SA (1.001 [1.000–1.002], *P* = 0.001), Cys-C (1.008 [1.006–1.009], *P* < 0.001,) and glucose (0.817 [0.682–0.978], *P* = 0.028) were independently associated with eCRS.

**Table 5:** Univariate and multivariate logistic regression analysis between eCRS patients and non-eCRS patients

Variables	Univariate analysis			Multivariable analysis^#^	
	OR (95% CI)	*P*		OR (95% CI)	*P*
Gender, male	1.638 (1.226–2.189)	< 0.001		1.148 (0.733–1.799)	0.507
Age, years	1.003 (1.003–1.022)	0.009		1.002 (0.990–1.013)	0.795
Smoking dependence	1.544 (1.191–2.001)	0.001		0.914 (0.624–1.340)	0.646
TG, mmol/L	1.220 (1.078–1.380)	< 0.001		1.121 (0.969–1.297)	0.125
FFA, mmol/L	0.315 (0.170–0.584)	< 0.001		0.306 (0.142–0.658)	0.002
LP(a), mg/L	1.000 (0.999-1.000)	0.179		—	—
IBIL, µmol/L	1.016 (0.991–1.041)	0.205		—	—
SA, mg/L	1.001 (1.000-1.002)	< 0.001		1.001 (1.000-1.002)	0.001
Crea, µmol/L	1.016 (1.008–1.024)	< 0.001		0.997 (0.987–1.007)	0.582
Cys-C, mg/mL	1.008 (1.006–1.009)	< 0.001		1.008 (1.006–1.009)	< 0.001
Glucose, mmol/L	0.819 (0.704–0.952)	0.009		0.817 (0.682–0.978)	0.028
UA, µmol/L	1.002 (1.001–1.004)	< 0.001		1.001 (0.999–1.003)	0.349

^#^ BMI and Alcohol dependence were adjusted as covariates. ADA, adenosine deaminase; CI, confidence intervals; Crea, creatinine; Cys-C, cystatin C; eCRS, eosinophilic chronic rhinosinusitis; FFA, free fatty acid; IBIL, Indirect bilirubin; LP(a), lipoprotein(a); non-eCRS, non-eosinophilic chronic rhinosinusitis; OR, odds ratio; SA, sialic acid; TG, triglyceride; UA, uric acid

**Fig. 2:**
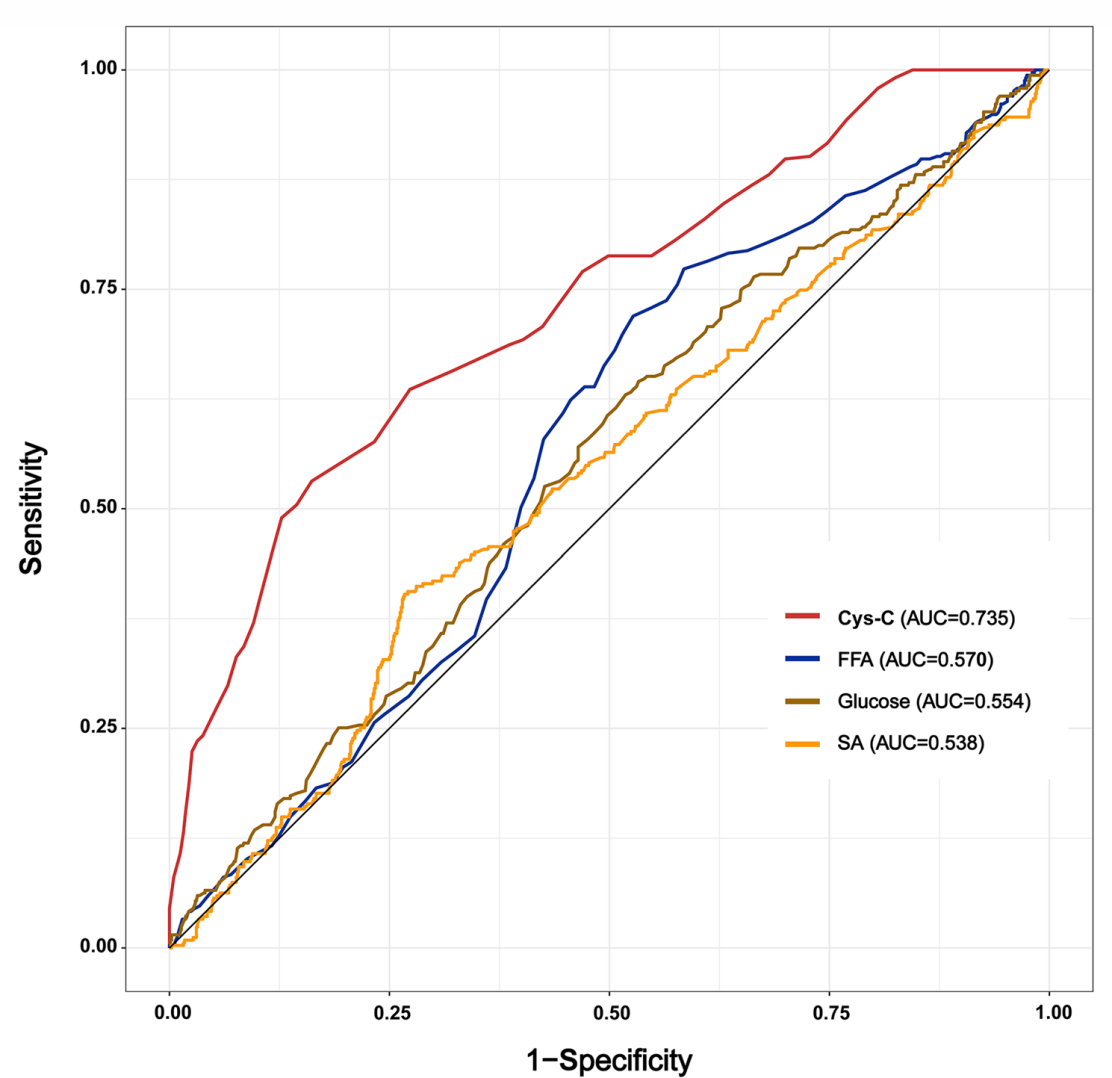
Receiver operating characteristic curves of peripheral blood metabolic indices for predicting eosinophilic chronic rhinosinusitis. Cys-C, cystatin C; FFA, free fatty acid; SA, sialic acid

ROC curves for predicting eCRS were established using FFA, SA, Cys-C, and glucose as independent predictors (Fig. 2), and the areas under the curve (AUC) of FFA, SA, Cys-C, and glucose were 0.570, 0.538, 0.735, and 0.554, respectively, confirming that the peripheral blood Cys-C had moderate ability to predict eCRS. The diagnostic cut-off value for Cys-C in predicting eCRS was determined to be 895 mg/mL, which yielded a sensitivity of 0.582, a specificity of 0.838, and a maximal Youden’s index of 0.420.

### Subgroup analysis and causal mediation analysis of eCRS patients and healthy controls

As shown in Table S2, the eCRS group had significantly higher age (*P* = 0.004), BMI (*P* < 0.001), and percentage of males (*P* < 0.001) and smoking dependence (*P* < 0.001) compared to the healthy control group, with no statistically significant distinction in alcohol dependence (*P* > 0.05).

Table S3 exhibits the subgroup analysis of peripheral blood metabolites. The levels of TG (*P* < 0.001), FFA (*P* < 0.001), LP(a) (*P* = 0.010), Crea (*P* < 0.001), Cys-C (*P* < 0.001), and UA (*P* < 0.001) were significantly higher in the eCRS group compared to the healthy control group; while LDH levels (*P* < 0.001) were significantly lower. No significant differences were observed in the levels of the other metabolites (*P* > 0.05).

Statistically significant variables were included in the univariate logistic regression analysis, and the results are shown in Table S4, gender (OR [95% CI]: 2.847 [2.138–3.790], *P* < 0.001), age (1.014 [1.003–1.026], *P* = 0.014), BMI (1.208 [1.157–1.261], *P* < 0.001), smoking dependence (1.360 [1.051–1.761], *P* = 0.019), TG (1.273 [1.128–1.435], *P* < 0.001), FFA (8.777 [4.745–16.233], *P* < 0.001), LDH (0.992 [0.987–0.996], *P* < 0.001), Crea (1.020 [1.013–1.028], *P* < 0.001), Cys-C (1.009 [1.007–1.010], *P* < 0.001), and UA (1.006 [1.005–1.007], *P* < 0.001) were associated with eCRS. The above associated variables were entered into a multivariate logistic regression model, with adjustment for alcohol dependence as covariate, the results showed that gender (2.648 [1.626–4.432], *P* < 0.001), BMI (1.181 [1.115–1.251], *P* < 0.001), smoking dependence (0.555 [0.368–0.839], *P* = 0.005),FFA 11.948 [5.413–26.375], *P* < 0.001), LDH (0.986 [0.980–0.991], *P* < 0.001), and Cys-C (1.008 [1.007–1.010], *P* < 0.001) were independently associated with eCRS.

**Fig. 3:**
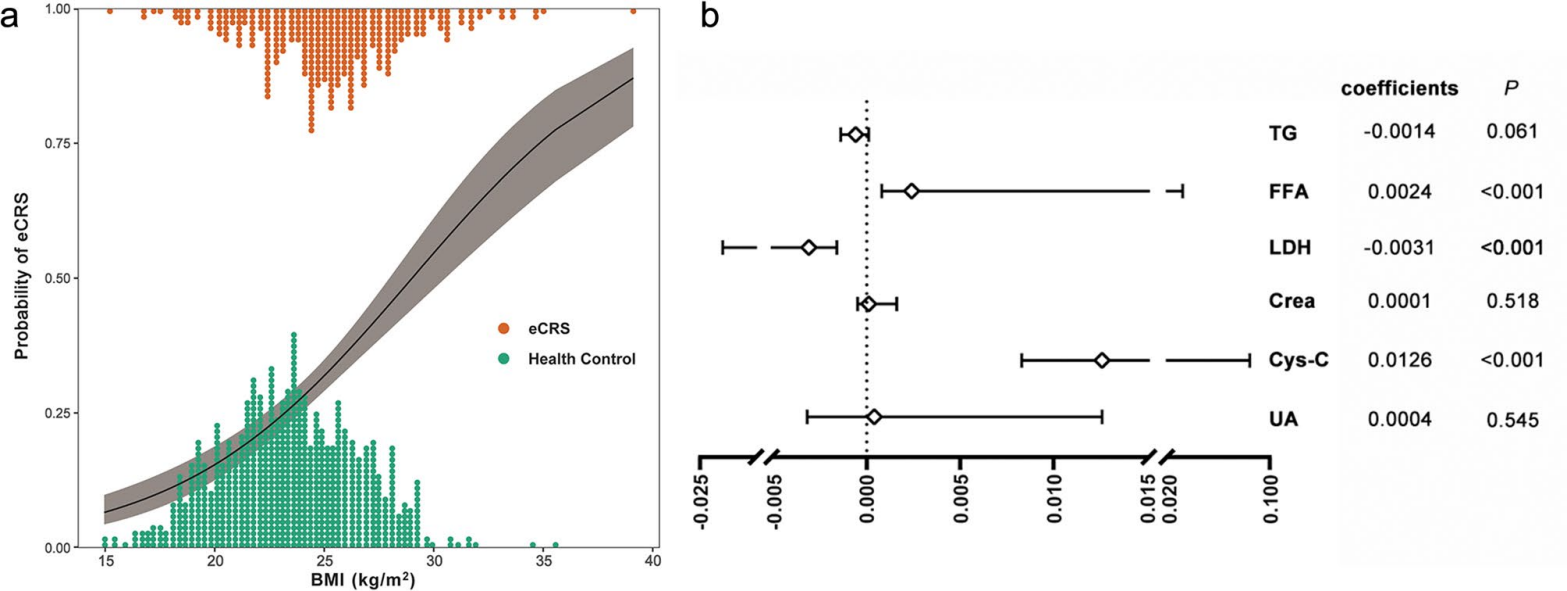
Relationship between BMI and eCRS and analysis of causal mediating effects. **a** Logistic regression fitted curve for BMI and risk of eCRS. **b** Causal mediation effect analysis of peripheral blood metabolic indices mediating the correlation between BMI and eCRS. BMI, body mass index; Crea, creatinine; Cys-C, cystatin C; eCRS, eosinophilic chronic rhinosinusitis; FFA, free fatty acid; LDH, lactate dehydrogenase; UA, uric acid

Figure 3a presented the logistic regression fitted curve for BMI and eCRS, demonstrating that the risk of eCRS increases with rising BMI.

Causal mediation models were used to explore the mediating effects of TG, FFA, LDH, Crea, Cys-C, and UA between BMI and eCRS. Gender, age, smoking dependence, and alcohol dependence were adjusted as covariates to further control for confounders, and the results are shown in Fig. 3b and Table S5, FFA (coefficients: 0.0024, 95% CI: 0.0008 to 0.0311, *P* < 0.001), LDH (coefficients: -0.0031, 95% CI: -0.0229 to -0.0016, *P* < 0.001), and Cys-C (coefficients: 0.0126, 95% CI: 0.0083 to 0.0842, *P* < 0.001) mediated the effect of BMI on eCRS.

### Subgroup analysis and causal mediation analysis of non-eCRS patients and healthy controls

As shown in Table S6, the non-eCRS group had a significantly higher percentage of males (*P* < 0.001) and BMI (*P* < 0.001) compared to the healthy control group, whereas no significant distinctions were noted for age, smoking dependence, or alcohol dependence (all *P* > 0.05).

Table S7 presents the subgroup analysis of peripheral blood metabolites. The non-eCRS group exhibited significantly higher levels of TG (*P* = 0.003), FFA (*P* < 0.001), LP(a) (*P* = 0.001), GLB (*P* = 0.037), Cys-C (*P* < 0.001), and UA (*P* < 0.001) compared to the healthy controls; while LDH levels were significantly lower (*P* < 0.001). The two groups showed no significant variation in the levels of the other metabolites (*P* > 0.05).

The univariate logistic regression, with results presented in Table S8, found that gender (OR [95% CI]: 1.738 [1.423–2.122], *P* < 0.001), BMI (1.162 [1.127–1.199], *P* < 0.001), FFA (26.200 [15.628–43.924], *P* < 0.001), LP(a) (1.001 [1.001–1.002], *P* < 0.001), GLB (1.018 [1.002–1.035], *P* = 0.032), LDH (0.995 [0.992–0.998], *P* = 0.001), Cys-C (30.109 [14.490–62,564], *P* < 0.001), UA (1.004 [1.003–1.005], *P* < 0.001) were all associated with non-eCRS. With adjustment for age, smoking dependence, and alcohol dependence, as covariates, multivariate logistic regression model showed that gender (OR [95% CI]: 2.560 [1.823–3.596], *P* < 0.001), BMI (1.155 [1.114–1.198], *P* < 0.001), FFA (21.021 [13.209–35.195], *P* < 0.001), LP(a) (1.019 [1.000–1.039], *P* < 0.001), GLB (1.001 [1.001–1.002], *P* = 0.046), LDH (0.990 [0.986–0.993], *P* < 0.001), Cys-C (1.002 [1.002–1.003], *P* < 0.001) were independently associated with non-eCRS.

**Fig. 4:**
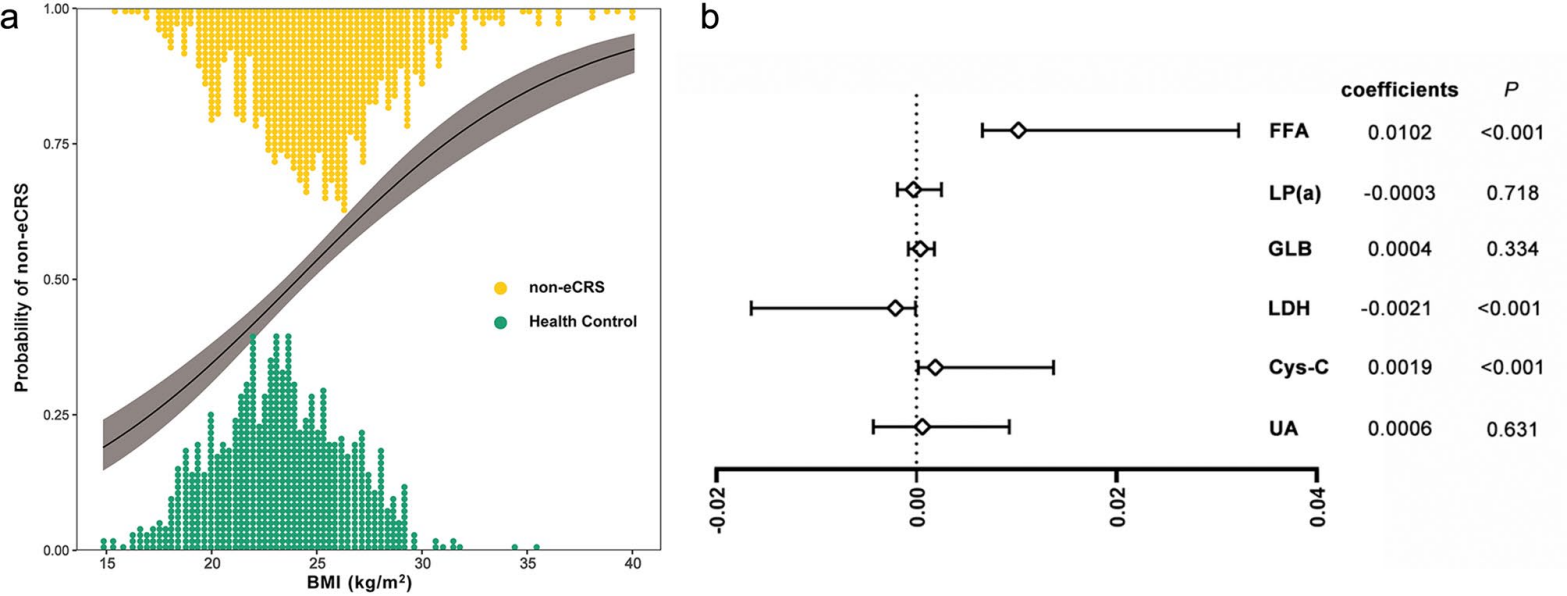
Relationship between BMI and non-eCRS and analysis of causal mediating effects. **a** Logistic regression fitting curve for BMI and risk of non-eCRS. **b** Causal mediation effect analysis of peripheral blood metabolic indices mediating the correlation between BMI and non-eCRS. BMI, body mass index; Cys-C, cystatin C; FFA, free fatty acid; GLB, globulin; LDH, lactate dehydrogenase; Lp(a), lipoprotein(a); non-eCRS, non-eosinophilic chronic rhinosinusitis; UA, uric acid

Figure 4a illustrates the logistic regression fitted curve for BMI and non-eCRS, demonstrating that the risk of non-eCRS increases with rising BMI.

Causal mediation models were used to explore the mediating effects of FFA, LP(a), GLB, LDH, Cys-C, and UA between BMI and non-eCRS. Gender, age, smoking dependence, and alcohol dependence were adjusted as covariates to further control for confounders, and the results are shown in Fig. 4b and Table S9, FFA (coefficients: 0.0102, 95% CI: 0.0066–0.0322, *P* < 0.001), LDH (coefficients: -0.0021, 95% CI: -0.0165–-0.0001, *P* < 0.001), Cys-C (coefficients: 0.0019, 95% CI: 0.0002–0.0137, *P* < 0.001) mediated the effect of BMI on non-eCRS.

## Discussion

This study investigated the peripheral blood metabolic profiles of different endotypes of CRS, revealing distinct patterns of metabolic abnormalities between eCRS and non-eCRS. These findings suggest that peripheral blood Cys-C is a promising biomarker for differentiating eCRS from non-eCRS. Furthermore, the results indicated that FFA and Cys-C mediated the effect of BMI on CRS in both eCRS and non-eCRS populations.

As individualized prevention and precision medicine gain prominence, the association between CRS and systemic metabolism has drawn increasing attention. Previous studies initially reported a potential correlation between CRS and metabolic syndrome [5, 6, 20]. In the present study, the differences in blood metabolic profiles between patients with CRS and healthy controls were assessed comprehensively, revealing that lipid metabolism abnormalities, particularly TG, FFA, and LP(a), were characteristic of both eCRS and non-eCRS. Multivariate logistic regression analyses demonstrated that elevated FFA represented an independent risk factor for both eCRS and non-eCRS populations. Interestingly, FFA was also found has also been implicated as an independent risk factor for newly-onset asthma in a recent study [21]; and CRS and asthma are considered to be highly homogeneous in pathophysiology based on the theory of “united airways diseases” [22]. Notably, eCRS patients exhibited higher TG levels, while non-eCRS patients had higher FFA levels, indicating distinct lipid metabolism patterns of the two endotypes, suggesting that TG may be implicated in the pathogenesis of type 2 inflammation, whereas FFA appears more involved in non-type 2 and mixed inflammation.

In addition to lipid metabolism abnormalities, this study also found elevated levels of Cys-C and UA in CRS patients, with higher levels observed in the eCRS group, suggesting that they may play stronger roles in type 2 inflammatory pathways than to their non-type 2 counterparts. Several previous studies have reported elevated peripheral blood Cys-C and UA in patients with asthma [23–26], but this appears to be the first report of such elevations in CRS patients compared to healthy controls. Elevated blood Cys-C and UA were risk factors for asthma, with higher levels in patients with poorly controlled asthma; Xie et al. [27] identified elevated UA as a risk factor for recurrence of CRS with nasal polyps. Given that eCRS is associated with type 2 inflammation and a higher risk of recurrence [28, 29], the results of this study showed strong concordance with the aforementioned studies, suggesting that elevated Cys-C and UA may be systemic markers of type 2 inflammation in CRS.

In recent years, discriminating between eCRS and non-eCRS has become a focus of research due to their significant differences in pathophysiology and clinical manifestations [30]. Despite belonging to the same disease spectrum, eCRS is generally more sensitive to glucocorticoids, yet manifests with a greater symptom burden and an increased propensity for recurrence, making it a refractory subtype in clinical practice [31–33]. Researchers are continuously exploring simple and convenient methods to differentiate eCRS from non-eCRS in clinical practice [34–36], but this remains a challenging task. This study identified Cys-C as an independent predictor of CRS endotypes, and it showed good predictive ability in the ROC curve as a single predictor. While previous studies have focused on cysteine proteases such as CST1 in eCRS pathogenesis [37–39], the current work is the first to document the predictive utility of Cys-C, one of the peripheral blood cysteine proteases, for eCRS, thereby offering a novel benchmark for endotypic classification of the disease. In China, Cys-C testing is routinely incorporated into the preoperative assessment of CRS patients, ensuring that it does not incur additional medical costs; moreover, this test is widely available in most healthcare institutions around the world. Cys-C is expected to be included as a predictive factor in the establishment of the composite prediction model for eCRS in the future, which can provide references for the selection of drugs, planning of surgical and follow-up strategies for patients with CRS.

The diagnostic cut-off value for eCRS remains controversial, with reported thresholds ranging from 5 to 350 eosinophils per HPF [40]. While the EPOS 2020 guidelines [3] recommend a cut-off value of 10/HPF, this study adopted 55/HPF based on two principal considerations. First, the EPOS recommendation primarily derives from the American population study by Soler et al. [41], however, given that factors such as ethnicity, geography, and environment can significantly affect this threshold, it is unlikely that a single cut-off value is universally applicable. Japanese studies [42–44] have typically employed a cut-off value of 70/HPF as established by the Japanese JESREC study [45]. Notably, Lou et al. [17] identified 55/HPF as the optimal threshold for northern Chinese populations, aligning with the demographic characteristics of the cohort in this study. Second, compared to subjective quality-of-life measures, disease recurrence as an objective clinical endpoint provides the most clinically significant and least biased parameter for threshold determination [40]. This cut-off value has been validated in a meta-analysis [28] as demonstrating superior diagnostic accuracy.

Several observational studies reported the association between BMI and CRS [10–12], and a prior MR study using GWAS data confirmed the unidirectional causal effect of BMI on CRS [13]. However, limited by disease typing of GWAS data, the previous study failed to differentiate endotypes of CRS; therefore, this study remedied the previous limitation and found that both eCRS and non-eCRS disease risk were significantly elevated with increasing BMI. Furthermore, causal mediation analysis revealed that peripheral blood FFA and Cys-C partially mediated the effect of BMI on both eCRS and non-eCRS, suggesting that dietary lifestyle interventions, weight control, and lipid-lowering therapies should be included as therapeutic considerations for patients with CRS, especially those with refractory CRS. In addition, partial mediating effects meant that BMI was also engaged in the pathogenesis of CRS through other pathways, which may be related to the obesity-induced state of systemic low-grade inflammation [46], and leptin, lipocalin, and other obesity-associated inflammatory factors [47].

### Study strengths and limitations

The present study is distinguished by several key strengths. First, the large-scale cohort, encompassing 1,151 CRS patients and 814 healthy controls, provides substantial statistical power to the findings. Second, this is the first reports to comprehensively compare peripheral metabolic profiles across CRS endotypes and healthy individuals, successfully identifying Cystatin-C as a novel and clinically accessible biomarker for differentiating eCRS from non-eCRS. A key methodological strength is the application of causal mediation analysis, which moves beyond simple association to offer deeper mechanistic insights into how metabolites mediate the established causal link between BMI and CRS.

There are still the following limitations. First, this study is a single-center, large-sample retrospective study based on the Chinese population, and further multicenter studies, especially involving other ethnic populations, are needed to enhance the generalizability of these findings. Second, given the changes in peripheral blood metabolic indexes during the developmental process, we included only adult CRS patients, limiting the applicability of the findings to children. Lastly, as a clinical study, an exploration of the underlying molecular mechanisms was beyond our scope. Elucidating these pathways should be a key objective for future basic science research.

## Conclusions

In conclusion, this study reveals that CRS has distinct peripheral metabolic profiles, with unique signatures for its eCRS and non-eCRS endotypes. A key clinical implication of this study is that Cys-C serves as a practical predictor for eCRS, guiding earlier risk stratification and personalized therapies. Furthermore, evidence that metabolites mediate obesity’s impact on CRS supports integrating weight management into holistic patient care, promoting preventive strategies. This work thus provides a foundation for validating these biomarkers and for mechanistic studies to understand how these metabolic changes drive sinonasal inflammation, potentially identifying novel therapeutic targets.

## Electronic supplementary material

Below is the link to the electronic supplementary material.

Supplementary Material 1

## Data Availability

The data are available from the corresponding author on reasonable request.

## Abbreviations

ADA: Adenosine deaminase
ALB: Albumin
ALT: Alanine aminotransferase
AST: Aspartate aminotransferase
AUC: Area under the curve
BMI: Body mass index
CI: Confidence interval
Crea: Creatinine
CRS: Chronic rhinosinusitis
Cys-C: Cystatin C
DBIL: Direct bilirubin
eCRS: Eosinophilic chronic rhinosinusitis
FFA: Free fatty acid
GGT: Gamma-glutamyl transferase
GLB: Globulin
GWAS: Genome-wide association studies
HDL: High-density lipoprotein
HPF: High-powered microscope field
IBIL: Indirect bilirubin
LAP: Leucine aminopeptidase
LDH: Lactate dehydrogenase
LDL: Low-density lipoprotein
LP(a): Lipoprotein(a)
MR: Mendelian randomization
non-eCRS: Non-eosinophilic chronic rhinosinusitis
OR: Odds ratio
PAB: Prealbumin
ROC: Receiver operating characteristic
SA: Sialic acid
TC: Total cholesterol
TG: Triglyceride
UA: Uric acid
