# Supplementary material for: Peripheral blood metabolic profiles of chronic rhinosinusitis and their mediating role between obesity and disease
Source: Lipids Health Dis. 2025 Jul 28;24:251. doi: 10.1186/s12944-025-02672-w (PMC12306000; doi:10.1186/s12944-025-02672-w)
Supplement: Supplementary file 1 — Supplementary Material 1 [file 12944_2025_2672_MOESM1_ESM.docx]

**Supplementary Materials**

Table S1 Univariate and multivariate logistic regression analysis between CRS patients and healthy controls

Table S2 Baseline characteristics of the eCRS patients and healthy controls

Table S3 Metabolic characteristics of peripheral blood between eCRS patients and healthy controls

Table S4 Univariate and multivariate logistic regression analysis between eCRS patients and healthy controls

Table S5 Causal mediation analysis of metabolites on peripheral blood for the associations between BMI and eCRS

Table S6 Baseline characteristics of the non-eCRS patients and healthy controls

Table S7 Metabolic characteristics of peripheral blood between eCRS patients and non-eCRS patients

Table S8 Univariate and multivariate logistic regression analysis between non-eCRS patients and healthy controls

Table S9 Causal mediation analysis of metabolites on peripheral blood for the associations between BMI and non-eCRS

Figure S1 Logistic regression fitted curve for BMI and risk of CRS.

**Table S1 Univariate and multivariate logistic regression analysis between CRS patients and healthy controls**

| Variables | Univariate analysis | |  | Multivariable analysis^#^ | |
| --- | --- | --- | --- | --- | --- |
|  | OR (95% CI) | *P* |  | OR (95% CI) | *P* |
| Gender, male | 1.986 (1.649-2.392) | <0.001 |  | 2.333 (1.678-3.245) | <0.001 |
| BMI, kg/m^2^ | 1.170 (1.137-1.204) | <0.001 |  | 1.156 (1.116-1.197) | <0.001 |
| TG, mmol/L | 1.148 (1.040-1.267) | 0.006 |  | 0.843 (0.750-0.947) | 0.004 |
| FFA, mmol/L | 22.803 (13.977-37.203) | <0.001 |  | 19.786 (11.557-33.874) | <0.001 |
| LP(a), mg/L | 1.001 (1.000-1.001) | <0.001 |  | 1.001 (1.000-1.002) | <0.001 |
| LDH, U/L | 0.994 (0.991-0.997) | <0.001 |  | 0.989 (0.985-0.992) | <0.001 |
| Crea, μmol/L | 1.009 (1.004-1.014) | <0.001 |  | 1.004 (0.997-1.011) | 0.221 |
| Cys-C, mg/mL | 1.005 (1.004-1.005) | <0.001 |  | 1.004 (1.003-1.005) | <0.001 |
| UA, μmol/L | 1.005 (1.004-1.006) | <0.001 |  | 1.001 (1.000-1.003) | 0.163 |

^#^ Age, smoking dependence and alcohol dependence were adjusted as covariate. BMI, body mass index; CI, confidence intervals; Crea, creatinine; CRS, eosinophilic chronic rhinosinusitis; Cys-C, cystatin C; FFA, free fatty acid; LDH, lactate dehydrogenase; LP(a), lipoprotein(a); OR, odds ratio; TG, triglyceride; UA, uric acid.

| **Table S2 Baseline characteristics of the eCRS patients and healthy controls** | | | |
| --- | --- | --- | --- |
|  | eCRS patients (n=335) | Health controls  (n=814) | *P* |
| Gender, male | 255 (76.12%) | 430 (52.83%) | <0.001 |
| Age, years^*^ | 53.00±18.00 | 51.00±14.00 | 0.004 |
| BMI, kg/m^2*^ | 25.20±4.30 | 23.16±4.22 | <0.001 |
| Smoking dependence | 150 (44.78%) | 304 (37.35%) | 0.019 |
| Alcohol dependence | 147 (43.88%) | 311 (38.21%) | 0.074 |

^*^ *M*±*Q_R_*; BMI, body mass index; eCRS, eosinophilic chronic rhinosinusitis.

| **Table S3 Metabolic characteristics of peripheral blood between eCRS patients and healthy controls** | | | |
| --- | --- | --- | --- |
|  | eCRS patients (n=335) | Health controls  (n=814) | *P* |
| TG, mmol/L^*^ | 1.29±1.00 | 1.04±0.94 | <0.001 |
| FFA, mmol/L^*^ | 0.40±0.23 | 0.31±0.27 | <0.001 |
| TC, mmol/L^*^ | 4.86±1.41 | 4.89±1.37 | 0.730 |
| LDL, mmol/L^*^ | 2.86±1.10 | 2.86±1.01 | 0.866 |
| HDL, mmol/L^*^ | 1.37±0.41 | 1.41±0.46 | 0.212 |
| LP(a), mg/L^*^ | 128.00±152.00 | 110.00±129.25 | 0.010 |
| ALB, g/L^*^ | 47.77±13.60 | 48.56±13.00 | 0.152 |
| GLB, g/L^*^ | 30.50±8.59 | 30.46±8.92 | 0.894 |
| PAB, mg/L^*^ | 287.80±97.20 | 283.90±85.40 | 0.393 |
| DBIL, μmol/L^*^ | 3.70±2.26 | 3.58±2.09 | 0.081 |
| IBIL, μmol/L^*^ | 10.00±4.88 | 10.02±4.67 | 0.192 |
| ALT, U/L^*^ | 19.10±14.00 | 19.70±13.45 | 0.585 |
| AST, U/L^*^ | 18.00±6.80 | 18.50±6.75 | 0.093 |
| GGT, U/L^*^ | 19.00±18.00 | 18.60±6.00 | 0.688 |
| LDH, U/L^*^ | 163.00±36.00 | 170.00±39.00 | <0.001 |
| LAP, U/L^*^ | 41.90±8.20 | 42.00±11.30 | 0.471 |
| ADA, U/L^*^ | 9.00±4.00 | 9.00±3.00 | 0.151 |
| SA, mg/L^*^ | 66.09±436.97 | 62.94±436.50 | 0.042 |
| Crea, μmol/L^*^ | 84.30±22.00 | 78.00±24.33 | <0.001 |
| Cys-C, mg/mL^*^ | 910±180 | 730±180 | <0.001 |
| C1q, mg/L^*^ | 173.00±50.50 | 177.00±47.90 | 0.260 |
| Glucose, mmol/L^*^ | 4.90±0.77 | 4.95±0.83 | 0.125 |
| UA, μmol/L^*^ | 355.20±127.00 | 296.00±125.00 | <0.001 |

^*^ *M*±*Q_R_*; BMI, body mass index; ADA, adenosine deaminase; ALB, albumin; ALT, alanine aminotransferase; AST, aspartate aminotransferase; Crea, creatinine; CRS, chronic rhinosinusitis; Cys-C, cystatin C; DBIL, direct bilirubin; FFA, free fatty acid; eCRS, eosinophilic chronic rhinosinusitis; GGT, gamma-glutamyl transferase; GLB, globulin; HDL, high-density lipoprotein; IBIL, Indirect bilirubin; LAP, leucine aminopeptidase; LDH, lactate dehydrogenase; LDL, low-density lipoprotein; LP(a), lipoprotein(a); PAB, prealbumin; SA, sialic acid; TC, total cholesterol; TG, triglyceride; UA, uric acid.

**Table S4 Univariate and multivariate logistic regression analysis between eCRS patients and healthy controls**

| Variables | Univariate analysis | |  | Multivariable analysis^#^ | |
| --- | --- | --- | --- | --- | --- |
|  | OR (95% CI) | *P* |  | OR (95% CI) | *P* |
| Gender, male | 2.847 (2.138-3.790) | <0.001 |  | 2.648 (1.626-4.432) | <0.001 |
| Age, years | 1.014 (1.003-1.026) | 0.014 |  | 0.996 (0.981-1.011) | 0.609 |
| BMI, kg/m^2^ | 1.208 (1.157-1.261) | <0.001 |  | 1.181 (1.115-1.251) | <0.001 |
| Smoking dependence | 1.360 (1.051-1.761) | 0.019 |  | 0.555 (0.368-0.839) | 0.005 |
| TG, mmol/L | 1.273 (1.128-1.435) | <0.001 |  | 0.853 (0.723-1.007) | 0.061 |
| FFA, mmol/L | 8.777 (4.745-16.233) | <0.001 |  | 11.948 (5.413-26.375) | <0.001 |
| LP(a), mg/L | 1.001 (1.000-1.001) | 0.071 |  | — | — |
| LDH, U/L | 0.992 (0.987-0.996) | <0.001 |  | 0.986 (0.980-0.991) | <0.001 |
| SA, mg/L | 1.000 (1.000-1.001) | 0.345 |  | — | — |
| Crea, μmol/L | 1.020 (1.013-1.028) | <0.001 |  | 1.003 (0.993-1.014) | 0.518 |
| Cys-C, mg/mL | 1.009 (1.007-1.010) | <0.001 |  | 1.008 (1.007-1.010) | <0.001 |
| UA, μmol/L | 1.006 (1.005-1.007) | <0.001 |  | 1.001 (0.998-1.003) | 0.545 |

^#^ Alcohol dependence was adjusted as covariate. ADA, adenosine deaminase; BMI, body mass index; CI, confidence intervals; Crea, creatinine; Cys-C, cystatin C; FFA, free fatty acid; eCRS, eosinophilic chronic rhinosinusitis; IBIL, Indirect bilirubin; LDH, lactate dehydrogenase; LP(a), lipoprotein(a); OR, odds ratio; SA, sialic acid; TG, triglyceride; UA, uric acid.

| **Table S5 Causal mediation analysis of metabolites on peripheral blood for the associations between BMI and eCRS** | | | | | |
| --- | --- | --- | --- | --- | --- |
|  |  |  |  | 95% CI for coefficients | |
| Mediating variables | coefficients | se | *P* | Lower | Upper |
| TG, mmol/L | -0.0014 | 0.0061 | 0.061 | -0.0232 | 0.0001 |
| FFA, mmol/L | 0.0024 | 0.0083 | <0.001 | 0.0008 | 0.0311 |
| LDH, U/L | -0.0031 | 0.0058 | <0.001 | -0.0229 | -0.0016 |
| Crea, μmol/L | 0.0001 | 0.0006 | 0.518 | -0.0005 | 0.0016 |
| Cys-C, mg/mL | 0.0126 | 0.0210 | <0.001 | 0.0083 | 0.0842 |
| UA, μmol/L | 0.0004 | 0.0037 | 0.545 | -0.0032 | 0.0126 |

BMI, body mass index; CI, confidence intervals; Crea, creatinine; Cys-C, cystatin C; FFA, free fatty acid; eCRS, eosinophilic chronic rhinosinusitis; LDH, lactate dehydrogenase; SA, sialic acid; se, standard error; TG, triglyceride; UA, uric acid.

| **Table S6 Baseline characteristics of the non-eCRS patients and healthy controls** | | | |
| --- | --- | --- | --- |
|  | non-eCRS patients (n=816) | Health controls  (n=814) | *P* |
| Gender, male | 539 (66.05%) | 430 (52.83%) | <0.001 |
| Age, years^*^ | 52.00±22.00 | 51.00±14.00 | 0.836 |
| BMI, kg/m^2*^ | 24.95±4.48 | 23.16±4.22 | <0.001 |
| Smoking dependence | 281 (34.44%) | 304 (37.35%) | 0.221 |
| Alcohol dependence | 310 (37.99%) | 311 (38.21%) | 0.928 |

^*^ *M*±*Q_R_*; BMI, body mass index; non-eCRS, non-eosinophilic chronic rhinosinusitis.

| **Table S7 Metabolic characteristics of peripheral blood between eCRS patients and non-eCRS patients** | | | |
| --- | --- | --- | --- |
|  | non-eCRS patients  (n=816) | Health controls  (n=814) | *P* |
| TG, mmol/L^*^ | 1.11±0.86 | 1.04±0.94 | 0.003 |
| FFA, mmol/L^*^ | 0.48±0.30 | 0.31±0.27 | <0.001 |
| TC, mmol/L^*^ | 4.88±1.45 | 4.89±1.37 | 0.581 |
| LDL, mmol/L^*^ | 2.83±1.04 | 2.86±1.01 | 0.106 |
| HDL, mmol/L^*^ | 1.40±0.45 | 1.41±0.46 | 0.766 |
| LP(a), mg/L^*^ | 125.00±174.40 | 110.00±129.25 | 0.001 |
| ALB, g/L^*^ | 48.52±12.36 | 48.56±13.00 | 0.081 |
| GLB, g/L^*^ | 30.88±8.24 | 30.46±8.92 | 0.037 |
| PAB, mg/L^*^ | 292.30±93.22 | 283.90±85.40 | 0.069 |
| DBIL, μmol/L^*^ | 3.60±2.09 | 3.58±2.09 | 0.899 |
| IBIL, μmol/L^*^ | 9.84±4.98 | 10.02±4.67 | 0.328 |
| ALT, U/L^*^ | 19.00±13.40 | 19.70±13.45 | 0.143 |
| AST, U/L^*^ | 18.00±7.00 | 18.50±6.75 | 0.145 |
| GGT, U/L^*^ | 19.00±18.50 | 18.60±6.00 | 0.467 |
| LDH, U/L^*^ | 163.00±36.80 | 170.00±39.00 | <0.001 |
| LAP, U/L^*^ | 42.00±10.68 | 42.00±11.30 | 0.152 |
| ADA, U/L^*^ | 9.00±4.00 | 9.00±3.00 | 0.817 |
| SA, mg/L^*^ | 63.48±403.09 | 62.94±436.50 | 0.612 |
| Crea, μmol/L^*^ | 79.00±26.00 | 78.00±24.33 | 0.166 |
| Cys-C, mg/mL^*^ | 800±180 | 730±180 | <0.001 |
| C1q, mg/L^*^ | 178.00±45.45 | 177.00±47.90 | 0.461 |
| Glucose, mmol/L^*^ | 5.03±0.89 | 4.95±0.83 | 0.079 |
| UA, μmol/L^*^ | 326.00±119.52 | 296.00±125.00 | <0.001 |

^*^ *M*±*Q_R_*; ADA, adenosine deaminase; ALB, albumin; ALT, alanine aminotransferase; AST, aspartate aminotransferase; Crea, creatinine; Cys-C, cystatin C; DBIL, direct bilirubin; FFA, free fatty acid; eCRS, eosinophilic chronic rhinosinusitis; GGT, gamma-glutamyl transferase; GLB, globulin; HDL, high-density lipoprotein; IBIL, Indirect bilirubin; LAP, leucine aminopeptidase; LDH, lactate dehydrogenase; LDL, low-density lipoprotein; LP(a), lipoprotein(a); non-eCRS, non-eosinophilic chronic rhinosinusitis; PAB, prealbumin; SA, sialic acid; TC, total cholesterol; TG, triglyceride; UA, uric acid.

**Table S8 Univariate and multivariate logistic regression analysis between non-eCRS patients and healthy controls**

| Variables | Univariate analysis | |  | Multivariable analysis | |
| --- | --- | --- | --- | --- | --- |
|  | OR (95% CI) | *P* |  | OR (95% CI) | *P* |
| Gender, male | 1.738 (1.423-2.122) | <0.001 |  | 2.560 (1.823-3.596) | <0.001 |
| BMI, kg/m^2^ | 1.162 (1.127-1.199) | <0.001 |  | 1.155 (1.114-1.198) | <0.001 |
| TG, mmol/L | 1.078 (0.968-1.200) | 0.171 |  | — | — |
| FFA, mmol/L | 26.200 (15.628-43.924) | <0.001 |  | 21.021 (13.209-35.195) | <0.001 |
| LP(a), mg/L | 1.001 (1.001-1.002) | <0.001 |  | 1.019 (1.000-1.039) | <0.001 |
| GLB, g/L | 1.018 (1.002-1.035) | 0.032 |  | 1.001 (1.001-1.002) | 0.046 |
| LDH, U/L | 0.995 (0.992-0.998) | 0.001 |  | 0.990 (0.986-0.993) | <0.001 |
| Cys-C, mg/mL | 30.109 (14.490-62.564) | <0.001 |  | 1.002 (1.002-1.003) | <0.001 |
| UA, μmol/L | 1.004 (1.003-1.005) | <0.001 |  | 1.000 (0.999-1.002) | 0.630 |

^#^ Age, smoking dependence, and alcohol dependence were adjusted as covariates. BMI, body mass index; CI, confidence intervals; Cys-C, cystatin C; FFA, free fatty acid; GLB, globulin; LDH, lactate dehydrogenase; LP(a), lipoprotein(a); non-eCRS, non-eosinophilic chronic rhinosinusitis; OR, odds ratio; TG, triglyceride; UA, uric acid.

| **Table S9 Causal mediation analysis of metabolites on peripheral blood for the associations between BMI and non-eCRS** | | | | | |
| --- | --- | --- | --- | --- | --- |
|  |  |  |  | 95% CI for coefficients | |
| Mediating variables | coefficients | se | *P* | Lower | Upper |
| FFA, mmol/L | 0.0102 | 0.0077 | <0.001 | 0.0066 | 0.0322 |
| LP(a), mg/L | -0.0003 | 0.0010 | 0.7181 | -0.0019 | 0.0025 |
| GLB, g/L | 0.0004 | 0.0006 | 0.334 | -0.0008 | 0.0018 |
| LDH, U/L | -0.0021 | 0.0050 | <0.001 | -0.0165 | -0.0001 |
| Cys-C, mg/mL | 0.0019 | 0.0043 | <0.001 | 0.0002 | 0.0137 |
| UA, μmol/L | 0.0006 | 0.0031 | 0.6305 | -0.0043 | 0.0093 |

BMI, body mass index; CI, confidence intervals; Cys-C, cystatin C; FFA, free fatty acid; GLB, globulin; LDH, lactate dehydrogenase; LP(a), lipoprotein(a); non-eCRS, non-eosinophilic chronic rhinosinusitis; se, standard error; UA, uric acid.


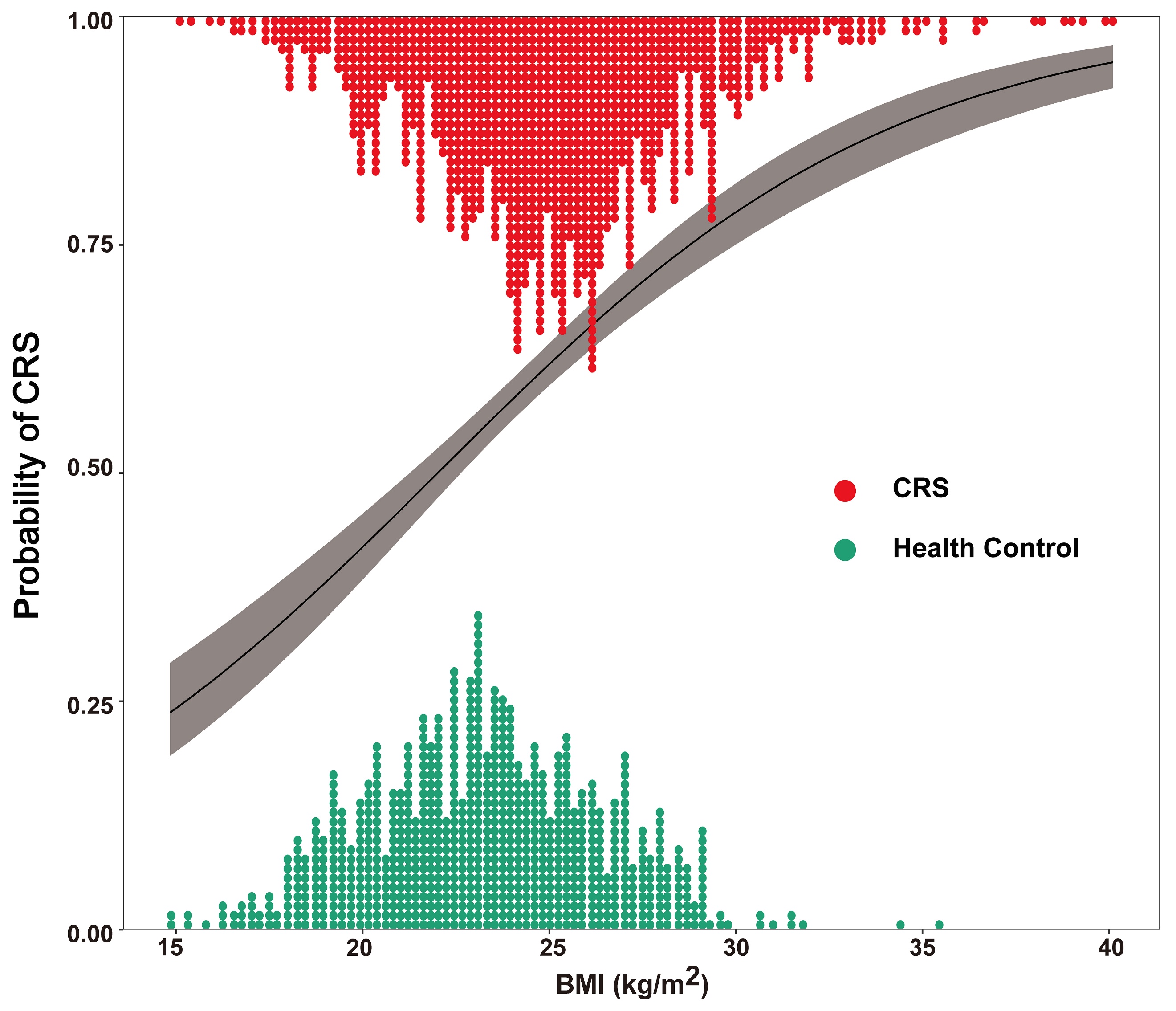
**Figure S1 Logistic regression fitted curve for BMI and risk of CRS.**

BMI, body mass index; CRS, chronic rhinosinusitis.
